# Acetylome analysis reveals the involvement of lysine acetylation in biosynthesis of antibiotics in *Bacillus amyloliquefaciens*

**DOI:** 10.1038/srep20108

**Published:** 2016-01-29

**Authors:** Lin Liu, Guangyuan Wang, Limin Song, Binna Lv, Wenxing Liang

**Affiliations:** 1 Shandong Province Key Laboratory of Applied Mycology, College of Life Sciences, Qingdao Agricultural University, Qingdao 266109, China; 2 The Key Laboratory of Integrated Crop Pest Management of Shandong Province, College of Agronomy and Plant Protection, Qingdao Agricultural University, Qingdao 266109, China

## Abstract

Lysine acetylation is a major post-translational modification that plays an important regulatory role in almost every aspects in both eukaryotes and prokaryotes. *Bacillus amyloliquefaciens*, a Gram-positive bacterium, is very effective for the control of plant pathogens. However, very little is known about the function of lysine acetylation in this organism. Here, we conducted the first lysine acetylome in *B. amyloliquefaciens* through a combination of highly sensitive immune-affinity purification and high-resolution LC−MS/MS. Overall, we identified 3268 lysine acetylation sites in 1254 proteins, which account for 32.9% of the total proteins in this bacterium. Till date, this is the highest ratio of acetylated proteins that have been identified in bacteria. Acetylated proteins are associated with a variety of biological processes and a large fraction of these proteins are involved in metabolism. Interestingly, for the first time, we found that about 71.1% (27/38) and 78.6% (22/28) of all the proteins tightly related to the synthesis of three types of pepketides and five families of lipopeptides were acetylated, respectively. These findings suggest that lysine acetylation plays a critical role in the regulation of antibiotics biosynthesis. These data serves as an important resource for further elucidation of the physiological role of lysine acetylation in *B. amyloliquefaciens*.

Nowadays the intensive use of agrochemicals has led to the emergence of pathogen resistance and severe negative environmental impacts^1^. With the increased demand for pesticide-free food, biological control through the use of natural antagonistic microorganisms has emerged as a promising alternative to chemical pesticides for more rational and safe crop management. *Bacillus amyloliquefaciens*, a Gram-positive bacterium, is highly effective for the biocontrol of multiple plant diseases caused by soilborne or post-harvest pathogens^2^,^3^,^4^,^5^,^6^. Especially, the strain FZB42 of *B. amyloliquefaciens* has been commercially used due to its high efficacy against fungal and bacterial pathogens^7^. Antibiotics of this organism play an important role in its biocontrol activity^8^. Lipopeptides (LPs) and polyketides, generated by complex enzymes known as non-ribosomal peptide synthetases (NRPS) and polyketide synthases (PKS), respectively^9^,^10^, are the best known antibiotics produced by *B. amyloliquefaciens*. These secondary metabolites not only suppress disease pressure in plants by antimicrobial activities and activating plant defense, but also are important for biofilm formation and root colonization of bacteria on crop plants^11^,^12^,^13^,^14^.

Protein acetylation is an evolutionarily conserved post-translational modification (PTM) in both eukaryotes and prokaryotes^15^. Through reversible addition of an acetyl group to lysine residues, protein acetylation regulates protein localization, activity, protein-protein and protein-nucleic acid interactions^16^. Advancements in mass spectrometry (MS) and high affinity purification of acetylated peptides have made it possible to study lysine acetylation on a proteomic scale. As a result, there is increasing evidence indicating that lysine acetylation is involved in diverse cellular processes, especially in regulating central metabolic pathways^17^,^18^,^19^,^20^,^21^,^22^,^23^,^24^. It was shown that in *Salmonella* the relative activities of key enzymes controlling the direction of glycolysis versus gluconeogenesis and the branching between citrate cycle and glyoxylate bypass were all regulated by acetylation^25^. However, to date, there is little information regarding the relationship between lysine acetylation and biologically active secondary metabolites production. The recent discoveries that several enzymes involved in the biosynthesis of secondary metabolites were found to be modified by acetyl groups implies a role of lysine acetylation in these processes^26^,^27^. In *Saccharopolyspora erythraea*, TDP-4-keto-6-deoxyhexose 2, 3-reductase and nonribosomal peptide synthetas, which are involved in the biosynthesis of erythromycin and siderophore, respectively, were identified as acetylated proteins. One NRPS involved in the production of unknown secondary metabolites in *Streptomyces roseosporus* was found to be acetylated on Lys703, a highly conserved residue in all NRPS adenylation domains^27^. Based on these observations, we speculate that lysine acetylation plays an important role in the regulation of the production of LPs and polyketides in *B. amyloliquefaciens*.

To test this hypothesis, we performed investigation on the acetylproteome of *B. amyloliquefaciens* by high-resolution LC-MS/MS coupled with highly sensitive immune-affinity purification. In total, we identified 3268 lysine acetylation sites in 1254 proteins, which account for 32.9% of the total proteins in the cell. The identified acetylated proteins are involved in diverse biological functions and cellular processes. Many enzymes associated with central carbon metabolism, protein metabolism and secondary metabolism are acetylated. Lysine acetylation is notably observed in the conserved domains of many key enzymes involved in the biosynthesis of polyketiedes and LPs, indicating that the biosynthesis of secondary metabolites could be regulated by acetylation. These results provide the first comprehensive view of the acetylome of *B. amyloliquefaciens*.

## Results

### Identification and analysis of lysine-acetylated proteins in *B. amyloliquefaciens*

**Figure 1:**
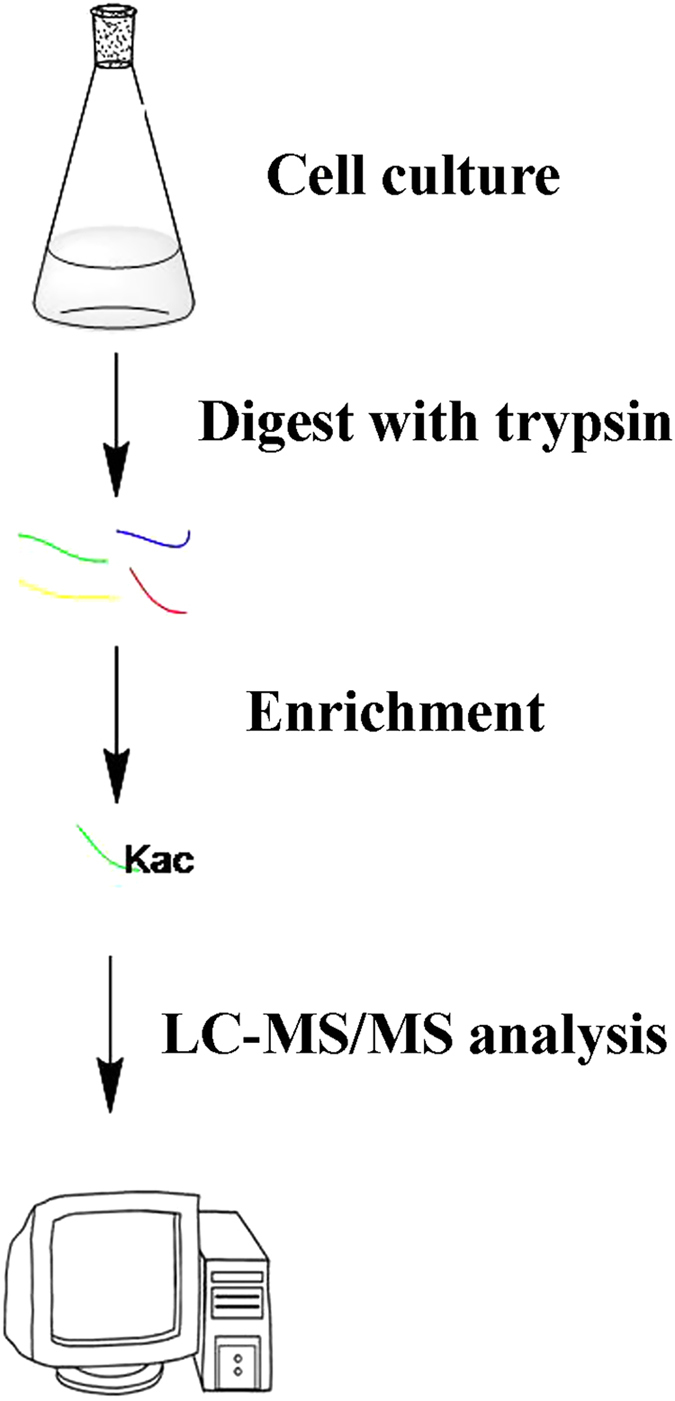
Overview of experimental procedures used in this study.

To determine the protein acetylome of *B. amyloliquefaciens*, a proteomic method based on sensitive immune-affinity purification and high-resolution LC-MS/MS was applied to identify acetylated proteins and their modification sites in *B. amyloliquefaciens* (Fig. 1). A total of 3268 lysine acetylation sites distributed in 1254 acetylated proteins was identified (Supplementary Table S1 online). The identified proteins account for 32.9% (1254/3811) of the total proteins in *B. amyloliquefaciens*, which is much higher than the percentage of acetylation reported in other bacteria (Table 1). To confirm the validation of MS data, the mass error of all the identified peptides were checked. The distribution of mass error is near zero and most of them are less than 0.02 Da which means the mass accuracy of the MS data fits the requirement (Supplementary Fig. S1a online). The length of most peptides was distributed between 8 and 20, which agrees with the property of tryptic peptides (Supplementary Fig. S1b online) and means sample preparation reaches the standard. The mass spectrometry proteomics data have been deposited to the ProteomeXchange Consortium via the PRIDE partner repository with the dataset identifier PXD003339.

### Functional annotation and cellular localization of acetylated proteins in *B. amyloliquefaciens*

**Figure 2:**
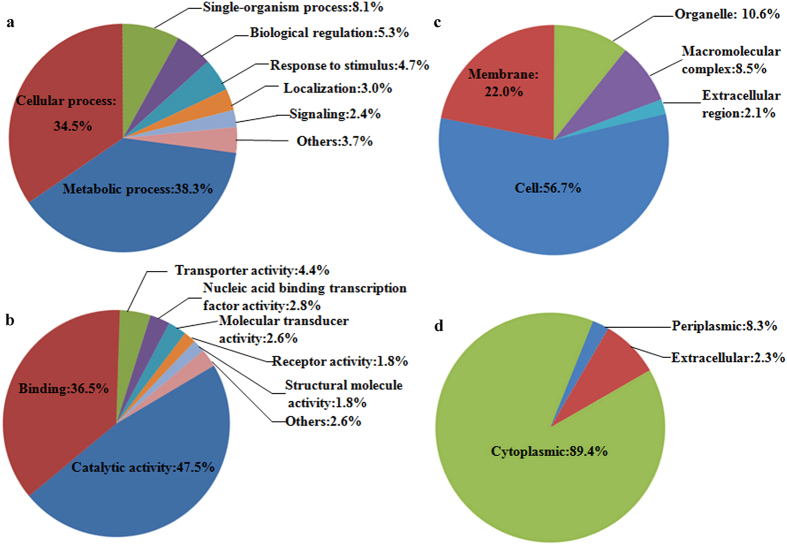
Pie charts showing the distribution of all the identified acetylated proteins. (**a**) Acetylated proteins categorized according to biological process. (**b**) Acetylated proteins categorized according to molecular function. (**c**) Acetylated proteins categorized according to cellular component. (**d**) subcellular localization.

To better understand the lysine acetylome in *B. amyloliquefaciens*, the Gene Ontology (GO) functional classification of all acetylated proteins based on their biological process, molecular function and cellular component was investigated (Fig. 2a–c, Supplementary Table S2 online). The GO analysis indicates that the acetylated proteins have diverse biological processes and molecular functions. Both of the results for biological process and molecular function showed that the largest group of acetylated proteins consists of enzymes associated with metabolism, which accounts for 38.3% and 47.5% of the total acetylated proteins, respectively (Fig. 2a,b). Another large acetyl protein group determined by biological process is associated with cellular processes, which accounts for 34.5% of all identified proteins. The second largest group in term of their molecular function is the set of binding proteins, and the number of proteins in this group is 36.5% of all modified proteins. The subcellular localization of the acetylated proteins was also analyzed, and the results showed that 1115 of the proteins were distributed in the cytoplasm (89.4%), 103 (8.3%) proteins were located to the periplasm, and 29 (2.3%) proteins were localized in the extracellular space (Fig. 2d).

### Analysis of acetylated lysine sites

**Figure 3:**
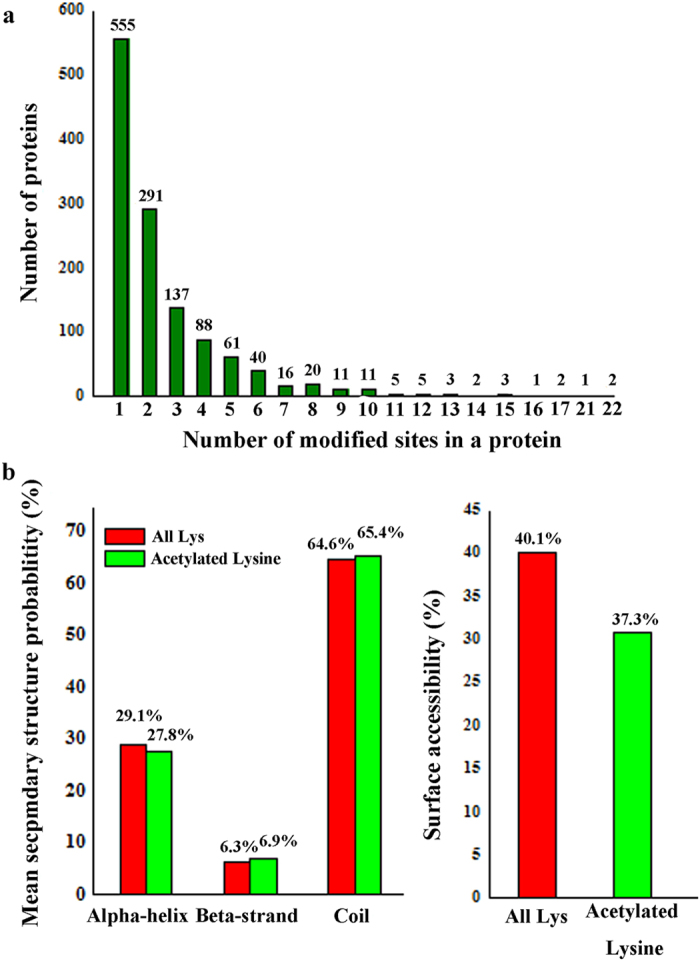
Properties of acetylated sites. (**a**) Distribution of acetylated sites in the acetylated proteins. (**b**) Probabilities of lysine acetylation in different protein secondary structures. (**c**) Predicted surface accessibility of acetylation sites.

A survey on the 3268 acetylated sites in the 1254 proteins showed that the number of lysine acetylation sites in each protein was from 1 to 22 (Fig. 3a). The average degree of acetylation was 2.6 sites per protein. More than 44% of the proteins contained only one acetylation site, whereas approximately 56% of the proteins were modified on multiple lysine residues. To explore the relationship between lysine acetylation and protein secondary structures, a structural analysis of all the acetylated proteins were performed. As shown in Fig. 3b, approximately 34.6% (1133) of the acetylation sites were located at regions with ordered secondary structure. Among them, 908 (27.8%) were distributed in α-helix and 225 (6.8%) sites were in β-strand. The remaining 65.4% (2135) acetylated sites were located in coils. However, the distribution pattern of the total lysine was very similar to that of the acetylated lysine, indicating there is no tendency of acetylation in *B. amyloliquefaciens*. This distribution pattern is similar to that of *Thermus thermophilus*^20^, but different from that of *Escherichia coli*^17^,^18^ and *Erwinia amylovora*^22^. Moreover, the enrichment of acetylated sites on the protein surface is very close to that of all lysine residues (Fig. 3c). Therefore, lysine acetylation is not likely to affect the surface property of modified proteins.

**Figure 4:**
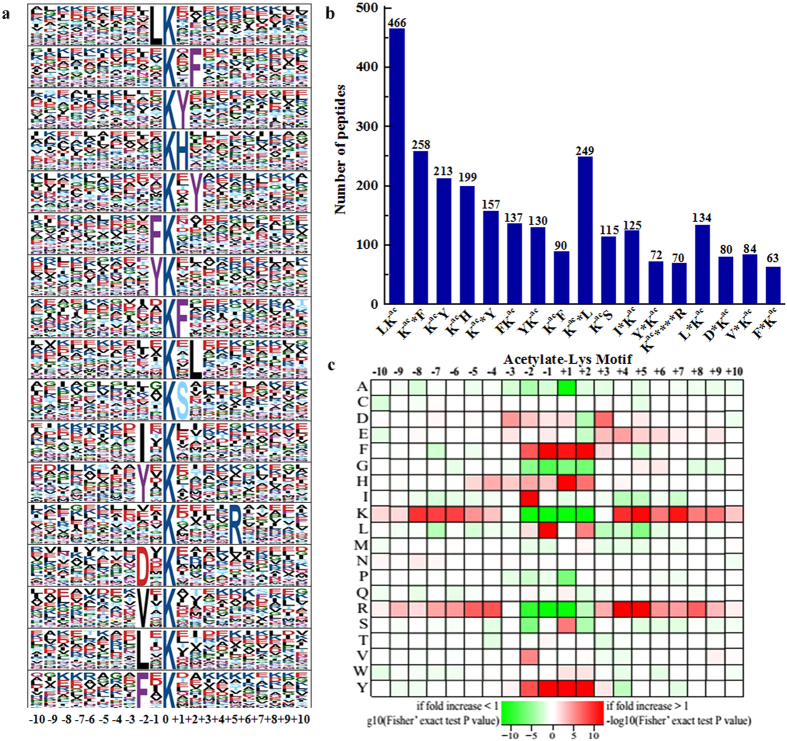
Properties of acetylated peptides. (**a**) Acetylation motifs and conservation of acetylation sites. (**b**) Number of identified peptides contained in each conserved motif. (**c**) Heat map of the amino acid compositions around the lysine acetylation sites showing the frequency of different types of amino acids surrounding this residue. Red indicates enrichment and green indicates depletion.

To further evaluate the nature of the acetylated lysines in *B. amyloliquefaciens*, the sequence motifs in all of the identified acetylated peptides were investigated using Motif-X program. 2642 acetylated peptides, which include the amino acid sequence from the −10 to +10 positions surrounding the acetylated lysine, were matched to seventeen conserved motifs (Fig. 4a). Particularly, motifs LK^ac^, K^ac^*F and K^ac^*L (K^ac^ indicates the acetylated lysine, and * represents a random amino acid residue) were strikingly conserved. The acetylated peptides with these motifs were 466, 258 and 249, which account for 17.6%, 9.8% and 9.4% of all the identified peptides, respectively (Fig. 4b, Supplementary Table S3 online). Importantly, although most of the identified motifs showed high frequency of occurrences in other prokaryotes and eukaryotes, the significantly conserved motifs, LK^ac^, K^ac^*F and K^ac^*L in *B. amyloliquefaciens*, were rarely identified in other organisms^17^,^18^,^19^,^20^,^21^,^22^,^23^,^24^. Given the fact that acetylated peptides with these three motifs account for 36.8% of all identified peptides, it is likely that proteins with these motifs have some special functions in *B. amyloliquefaciens*. As shown by the heat map of the amino acid compositions surrounding the acetylation sites, for all these motifs, the highest enrichment of a residue with aromatic groups, including tyrosine (Y) and phenylalanine (F), was observed in the −1 to +2 positions, whereas the occurrence frequency of a positively charged residue, lysine (K) and arginine (R), in positions −1 to +1 was the lowest (Fig. 4c). Therefore, proteins with Y or F, and without K or R in the corresponding positions would be the preferred substrates of lysine acetyltransferase in the cell. Moreover, the different preference of amino acid residues surrounding lysine sites suggests unique substrate preferences in *B. amyloliquefaciens*.

### Functional enrichment analysis

**Figure 5:**
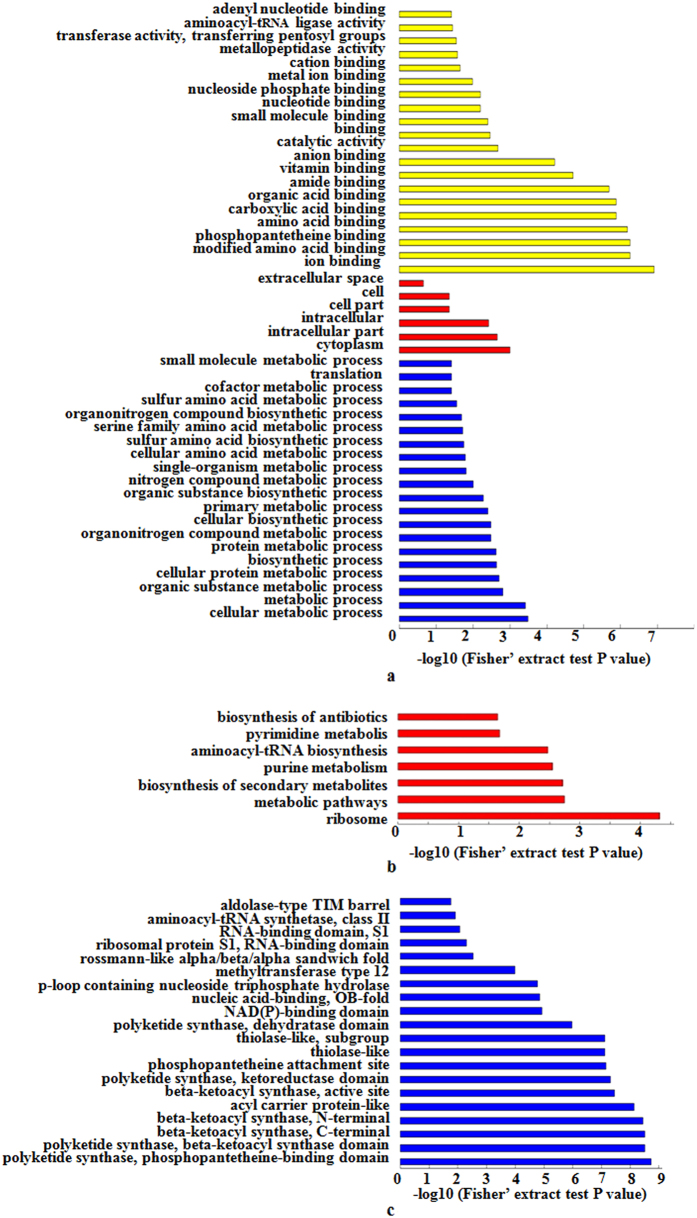
Enrichment analysis of the acetylated proteins in *B. amyloliquefaciens*. (**a**) A GO-based enrichment analysis of the acetylated proteins in terms of biological process (blue bars), molecular function (yellow bars) and cell component (red bars). (**b**) KEGG pathway enrichment analysis. (**c**) Protein domain enrichment analysis.

To determine which types of proteins are preferred targets for lysine acetylation, GO (biological process, molecular function, and cellular component), KEGG pathway and protein domain enrichment analyses were carried out. In the biological process category, most of the acetylated proteins were shown to be involved in metabolic and biosynthetic processes (Fig. 5a, Supplementary Table S4 online). In agreement with these observations, the enrichment analysis based on molecular function showed that many modified proteins were associated with binding and catalytic activity (Fig. 5a, Supplementary Table S5 online). Consistently, in the GO cellular component category, a large number of acetylated proteins were significantly enriched in the cytoplasm and intracellular space (Fig. 5a, Supplementary Table S6 online). Moreover, KEGG pathway enrichment analysis showed that a majority of the acetylated proteins were related to metabolic pathway and biosynthesis (Fig. 5b, Supplementary Fig. S2–S5 and Supplementary Table S7 online) such as biosynthesis of secondary metabolites and biosynthesis of antibiotics, implying an important regulatory role of lysine acetylation in these processes. To support these findings, polyketide synthase domain (including phosphopantetheine binding domain, ketoreductase domain and dehydratase domain), beta-ketoacyl synthase domain (including C-terminal, N-terminal and active site), acyl carrier protein-like domain, thiolase-like domain, phosphopantetheine attachment site domain and aminoacyl-tRNA synthetase domain (class II) were all highly enriched, as determined by protein domain enrichment analysis (Fig. 5c, Supplementary Table S8 online). All these domains are involved in the synthesis of LPs or polyketides, two important secondary metabolites in *B. amyloliquefaciens*.

### Acetylated proteins involved in biosynthesis of LPs and polypeptides

**Figure 6:**
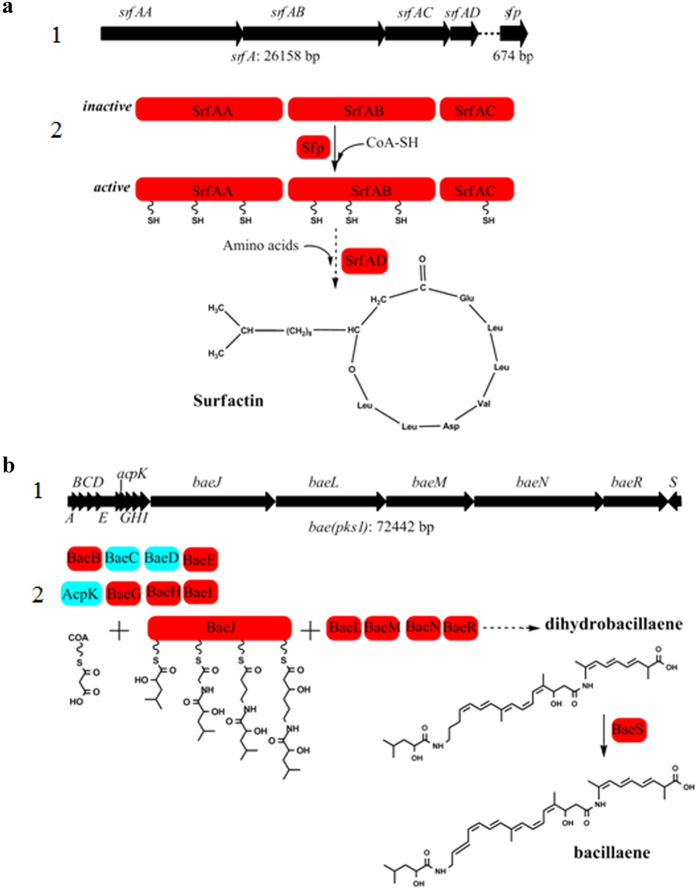
Biosynthsis of antibiotics in *B. amyloliquefaciens*. (**a**) Synthesis pathway of bacillaene, 1 gene cluster, 2 synthesis process. (**b**) Synthesis pathway of surfactin, 1 gene cluster, 2 synthesis process. The acetylated proteins are in red.

Notably, in this study, a total of 49 acetylated proteins were found to be involved in the synthesis of LPs and polyketides (Table 2, Fig. 6). The proteins related to the synthesis of five families of LPs, including surfactin, fengycin, putative peptide, bacillibactin and bacilysin, were acetylated (Table 2). Surprisingly, all the proteins in the synthesis pathway of surfactin (Srf AA, Srf AB, Srf AC, Srf AD and Sfp) and putative peptide (Nrs A, Nrs B, Nrs C, Nrs D, Nrs E and Nrs F), and most of proteins related to the biosynthesis of fengycin (Fen A, Fen B, Fen C and Fen D) and bacillibactin (Dhb A, Dhb B, Dhb C, Dhb E and Dhb F) were found to be acetylated. Proteins associated with the synthesis of three types of polyketide were also modified by acetyl groups. As shown in Table 2, most of the enzymes involved in the synthesis of macrolactin (Mln A, Mln B, Mln C, Mln D, Mln E, Mln F, Mln H and Mln I), bacillaene (Bae B, Bae E, Bae G, Bae H, Bae I, Bae J, Bae L, Bae M, Bae N, Bae R and Bae S) and difficidin (Dfn A, Dfn B, Dfn D, Dfn F, Dfn J, Dfn K, Dfn L and DfnY) were acetylated. Moreover, the majority of these acetylated proteins have multi acetylated sites, and many of the sites were distributed in the conserved domain of the proteins (Supplementary Table S9 online). For instance, the acetylated sites were observed in three core domains of PKS: the acyl transferase (AT) domain, the acyl carrier protein (ACP) domain and the ketosynthase (KS) domain. Acetylated sites were also found in other conserved domains including ketoreductases (KR), dehydratases (DH) and thioesterases (TE). In total, three domains in the mega-enzymes related to LPs synthesis were acetylated, which were the adenylation domain (in Nrs F, acetylated sites K269), the condensation domain (in Srf AC, acetylated sites K188; in Dhb F, acetylated sites K1085) and the thioesterase domain (in Srf AD, acetylated sites K6 and K98; Nrs B, acetylated sites K168). These data suggests that lysine acetylation may play a critical role in regulating the activity of these enzymes, and thus is important for the production of secondary metabolites in *B. amyloliquefaciens*.

## Discussion

Lysine acetylation is a dynamic, reversible and regulatory post-translational modification in both prokaryotes and eukaryotes with multiple functions^26^. Although *B. amyloliquefaciens* has been widely used for the biocontrol of a large number of plant diseases due to its ability to produce a vast array of biologically active secondary metabolites such as LPs and polyketides^28^,^29^, the role of lysine acetylation in the regulation of these processes is elusive. In this study, we determined the acetylome of *B. amyloliquefacien* with the identification of 3268 lysine acetylation sites in 1254 proteins. The acetylated proteins account for 32.9% of the total proteins in *B. amyloliquefaciens* proteome, which is dramatically higher than those previously reported in other bacteria. These modified protein are distributed in different cellular compartments and participate in a variety of biological processes. Importantly, a lot of proteins related to the biosynthesis of biologically active secondary metabolites were found to be acetylated. These findings probably uncover the crucial role of reversible acetylation in *B. amyloliquefaciens* and open up new possibilities for investigation in the field of secondary metabolism.

Consistent with previous contributions^17^,^18^,^19^,^20^,^21^,^22^, in *B. amyloliquefaciens* lysine acetylation is involved in multiple biological processes and molecular functions. The acetylproteome of *Bacillus subtilis* has been reported in 2013^19^, demonstrating that nearly all the enzymes associated with central metabolism including glycolysis, the TCA cycle, and the PPP were acetylated. Compared with *B. subtilis*, there is no significant difference in the variety of the acetylated proteins related to the central metabolism parthways. However, the acetylation level (1254, 32.9%) of *B. amyloliquefaciens* is markedly higher than that (185, 4.4%) in *B. subtilis*. These results suggest that in *B. amyloliquefaciens* lysine acetylation may be connected with some particular functions.

It is well known that *B. amyloliquefaciens* could produce various biologically active secondary metabolites, including LPs and polyketides. LPs and polyketides encompass a variety of cyclic, linear and branched structures and are synthesized by NPRS and PKS, respectively^30^,^31^. Notably, we found that 49 proteins involved in the synthesis of three types of polyketides (bacillaene, difficidin and macrolactin) and five types of LPs (surfactin, fengycin, bacillibactin, putative peptide and bacilysin) were acetylated. The acetylated proteins account for 71.1% (27/38) and 78.6% (22/28) of all the proteins in the synthesis pathway of pepketides and LPs, respectively. Bacillaene is a kind of polyketide with a linear structure comprising a conjugated hexaene^32^. Its biosynthesis has been described in *B. amyloliquefaciens* FZB42 and is encoded by a hybrid type I PKS-NPRS gene cluster called *bae*^29^. In total, there are 13 proteins involved in bacillaene synthesis. The protein Bae E was acetylated at K199 in the conserved AT domain that is responsible for activation and transfer of a simpler building unit (malonyl-CoA) to the ACP domain. Bae J was present with 22 acetylated sites. Among these sites, 4 (K37, K74, K543, K555) and 2 (K1358, K1511) were observed in the first and the second adenylation domain, which are responsible for the incorporation of α-hydroxy-isocaproic acid and glycine, respectively. There are 3 (K629, K1664, K3094) acetylated sites found in the phosphopantetheine attachment site of the ACP domain. A 4’-phosphopantetheine prosthetic group acts as a swinging arm for the attachment of activated fatty acid through a serine residue in the ACP domain. In Bae J, 1 site (K2040) in the KS domain (catalyzing decarboxylation and condensation reaction between the two ACP linked malonates), 4 sites (K3029, K4079, K4199, K4335) in the KR domain (catalyzing hydroxy group formation) and 2 sites (K2385, K2417) in the DH domain (forming double bonds after water elimination) were identified. In addition, an acetylated site of K2415 in Bae R was detected in the TE domain. Collectively, the acetylation sites were found to be distributed in almost every type of conserved domain in the acetylated proteins, indicating its important regulatory role in polyketides synthesis.

The gene clusters of the *Bacillus* encoding LPs such as surfactin, fengycin and bacillibactin have been described and summarized in detail^33^. NPRS comprise organized modules, and each module contains certain catalytic domains: the adenylation domain responsible for selection and monomer activation, the thiolation domain for transfer of the adenylated monomer to a NRPS bound PPT (phosphopantetheinyl transferase), the condensation domain for peptide bond formation and the thioesterase domain for release of the peptide monomer from NRPS. We found that essentially all the proteins related to the biosynthesis of surfactin, fengycin and bacillibactin were acetylated. Furthermore, some of the acetylated sites were located in the conserved domains of several mega-enzymes including Srf AC, Srf AD, Dhb F, Nrs B and Nrs F. These results indicate that lysine acetylation may play an important role in the regulation of the steps of adenylation, condensation and cyclization.

All these observations suggest that reversible acetylation plays a direct regulatory role in antibiotic synthesis in *B. amyloliquefaciens*. Lysine acetylation may affect the relative activities of enzymes in the synthesis pathways of LPs and polyketides and modulate metabolic flux for the biosynthesis of these secondary metabolites. The production of secondary metabolites is closely related to the nutrient levels and fermentation conditions^34^,^35^. For example, in the fermentation by *B. subtilis*, surfactin production in xylose, arabinose and glucose containing medium was 2700, 2600 and 2000 mg/L, respectively, whereas medium without any sugar showed low surfactin (700 mg/L) production^36^. In response to different nutrient conditions and the reaction environments, functional gene groups are regulated in diverse forms and at different levels^37^. Reversible acetylation of metabolic enzymes ensures that cells respond environmental changes via prompt sensing cellular energy status and flexible altering reaction rates or directions.

In conclusion, these results represent the first extensive data on lysine acetylation in *B. amyloliquefacien*. These data not only provides a rich resource for the examination of lysine acetylation in *B. amyloliquefacien*, but also expands our current knowledge of the regulatory function of acetylation in secondary metabolism.

## Methods

### Bacterial strain and culture

The *B. amyloliquefaciens* strain DSM 7 was grown in 80 mL of the Luria Bertani (LB) medium in 250 mL Erlenmeyer baffled flask by shaking at 180 rpm for 12 h at 37 ^o^C. Bacterial cells at exponential phase were harvested by centrifugation at 8000 rpm and 4 ^o^C for 5 min.

### Protein extraction and digestion

The centrifuged cell pellet was suspended in 8 M urea supplemented with 1% triton-100, 50 mM nicotinamide, 3 μM trichostatin A, 65 mM dithiothreitol (DTT) and 0.1% protease inhibitor cocktail III. The mixture was sonicated for 3 × 10 s at 20 s intervals, and the whole cell lysate was centrifuged at 12,000 rpm and 4 °C for 10 min to remove the cell debris. The protein in the supernatant was precipitated with 15% cold trichloroacetic acid for 2 h at −20 °C. The resulting precipitate was washed three times with ice-cold acetone. The protein was redissolved in buffer (pH 8.0) containing 8 M urea and 100 mM NH_4_CO_3_, and the protein concentration was determined with 2-D Quant kit (GE Healthcare) according to the manufacturer’s instructions. The protein sample was diluted by adding 100 mM NH_4_CO_3_ to urea concentration less than 2M and then digested with trypsin (Promega) at an enzyme-to-substrate ratio (1:50) at 37 ^o^C overnight. The tryptic peptides were reduced with 10 mM DTT for 1 h at 37 ^o^C and then alkylated with 20 mM iodoacetamide at room temperature for 45 min in darkness. The reaction was finally terminated with 15 mM cysteine for 30 min at room temperature. To ensure complete digestion, additional trypsin at an enzyme-to-substrate ratio (1:100) was added, and the mixture was incubated for an additional 4 h.

### Affinity enrichment of lysine acetylated peptide

The trypsin digested peptides were redissolved in NETN buffer (50 mM tris-HCl, 1 mM EDTA, 100 mM NaCl, 0.5% nonidet P-40, pH 8.0) and were then separated into 11 fractions by HPLC according to their hydrophilicity. Each fraction was incubated with pre-washed anti acetyllysine antibody agarose beads (PTM Biolabs) at 4 ^o^C overnight with gentle shaking. After incubation, the beads were washed four times with NETN buffer and twice with ddH_2_O. The bound peptides were eluted from the beads with 0.1% trifluoroacetic acid. The eluted fractions were combined and vacuum-dried. Prior to LC-MS/MS analysis, the obtained peptides were rinsed with C18 ZipTips (Millipore) according to the manufacturer’s instructions.

### LC-MS/MS analysis

The peptides were dissolved in 0.1% formic acid (FA) and the supernatant were directly loaded onto an reversed-phase pre-column (Acclaim PepMap 100, Thermo Scientific). Peptide separation was performed using a reversed-phase analytical column (Acclaim PepMap RSLC, Thermo Scientific). The gradient was comprised of an increase from 7% to 20% solvent B (0.1% FA in 98% acetonitrile) for 20 min, 20% to 35% for 8 min and climbing to 80% in 2 min then holding at 80% for the last 5 min, all at a constant flow rate of 300 nl/min on an EASY-nLC 1000 UPLC system. The resulting peptides were subjected to NSI source followed by tandem mass spectrometry (MS/MS) in Q Exactive^TM^ Plus hybrid quadrupole-Orbitrap mass spectrometer (Thermo Scientific) coupled online to the UPLC. Intact peptides were detected in the Orbitrap at a resolution of 70,000. Peptides were selected for MS/MS using NCE setting as 30. Ion fragments were detected in the Orbitrap at a resolution of 17,500. A data-dependent procedure that alternated between one MS scan followed by 20 MS/MS scans was applied for the top 20 precursor ions above a threshold ion count of 1E4 in the MS survey scan with 10.0s dynamic exclusion. The electrospray voltage applied was 2.0 kV. Automatic gain control (AGC) was used to prevent overfilling of the ion trap; 5E4 ions were accumulated for generation of MS/MS spectra. For MS scans, the m/z scan range was 350 to 1800. MS2 fixed first mass set as 100.

### Database Search

The protein and acetylation site identification was performed through MaxQuant with an integrated Andromeda search engine (v.1.4.1.2). The tandem mass spectra were searched against the UniProt_*B. amyloliquefaciens* database (28,235 sequences) concatenated with reverse decoy database. Trypsin was specified as cleavage enzyme allowing up to 4 missing cleavages, 5 modifications per peptide and 5 charges. The mass error was set to 10 ppm for precursor ions and 0.02 Da for fragment ions. Carbamidomethylation on Cys was specified as fixed modification, and oxidation on Met, acetylation on Lys, and acetylation on protein N-terminal were specified as variable modifications. False discovery rate thresholds for proteins, peptides and modification sites were specified at 1%. Minimum peptide length was set at 7. All the other parameters in MaxQuant were set to default values. The site localization probability was set as >0.75.

### Bioinformatics analyses

The GO annotation proteome was derived from the UniProt-GOA database (http://www.ebi.ac.uk/GOA). The proteins were classified by GO annotation based on three categories: biological process, molecular function and cellular component. The protein subcellular localization was analyzed with wolfpsort (an updated version of PSORT/PSORT II for the prediction of eukaryotic sequences). Secondary structures were predicted using NetSurfP. The Kyoto Encyclopedia of Genes and Genomes (KEGG) were used to annotate protein pathways. GO term, protein domain, and KEGG pathway enrichment were performed using the DAVID bioinformatics resources 6.7. Fisher’s exact test was used to test for the enrichment or depletion (two-tailed test) of specific annotation terms among members of resulting protein clusters. Correction for multiple hypothesis testing was carried out using standard false discovery rate control methods. Any terms with adjusted *p*-values below 0.05 in any of the clusters were treated as significant. Motif-X software was used to analyze the model of sequences constituted with amino acids in specific positions of acetyl-21-mers (10 amino acids upstream and downstream of the site) in all protein sequences. All the database protein sequences were used as background database parameter, other parameters with default values. For further hierarchical clustering based on categories, all the acetylation substance categories obtained after enrichment were first collated along with their *p* values, and then filtered for those categories which were at least enriched in one of the clusters with *p*-value < 0.05. This filtered *p*-value matrix was transformed by the function *x* = −log (*p* value), and the *x* values for each category were *z*-transformed. These *z* scores were then clustered by one-way hierarchical clustering (Euclidean distance, average linkage clustering) using Genesis. The cluster membership was visualized by a heat map using the “heatmap.2” function in the “gplot2” R-package.

## Additional Information

**How to cite this article**: Liu, L. *et al.* Acetylome analysis reveals the involvement of lysine acetylation in biosynthesis of antibiotics in *Bacillus amyloliquefaciens*. *Sci. Rep.* **6**, 20108; doi: 10.1038/srep20108 (2016).

## Supplementary Material

Supplementary Figures

Supplementary Table S1

Supplementary Table S2

Supplementary Table S3

Supplementary Table S4

Supplementary Table S5

Supplementary Table S6

Supplementary Table S7

Supplementary Table S8

Supplementary Table S9

## Figures and Tables

**Table 1:** Comparison of *B. amyloliquefaciens* acetylome with other published bacteria acetylomes.

Species	Total Proteins	Acetylated proteins	Percentage of acetylated proteins (%)	References
*B. amyloliquefaciens*	3811	1254	32.9	This study
*Vibrio parahemolyticus*	3079	656	21.3	38
*Mycobacterium tuberculosis*	4034	658	16.3	24
*Synechocystis sp*	3672	513	14.0	39
*Streptomyces roseosporus*	6315	667	10.6	27
*Escherichia coli*	4146	349	8.4	17
*Thermus thermophilus*	2238	128	5.7	20
*Pseudomonas aeruginosa*	5878	320	5.4	40
*Bacillus subtilis*	4176	185	4.4	19
*Salmonella enterica*	4525	191	4.2	25
*Geobacillus kaustophilus*	3653	114	3.1	21
*Erwinia amylovora*	3565	96	2.7	22

**Table 2:** The acetylated proteins involved in the synthesis of Lps and polyketides. The acetylated proteins are in bold.

Secondary metabolites	Lp or pepketide	Proteins in the synthesis pathways of Lps and pepketides	Percentage of acetylated proteins (%)
Lp	Surfactin	Srf AA, Sfr AB, Srf AC, Srf AD, Sfp	100.0%
	Fengycin	Fen A, Fen B, Fen C, Fen D, Fen E	80.0%
	Bacillibactin	Dhb A, Dhb B, Dhb C, Dhb D, Dhb E, Dhb F	83.3%
	Putative peptide	Nrs A, Nrs B, Nrs C, Nrs D, Nrs E, Nrs F	100.0%
	Bacilysin	Bac A, Bac B, Bac C, Bac D, BacE, Ywf G	33.3%
Pepketide	Bacillaene	Bae B, Bae C, Bae D, Bae E, Bae G, Bae H, Bae I, Bae J, Bae L, Bae M, Bae N, Bae R, Bae S, Acp K	78.6%
	Difficidin	Dfn A, Dfn B, Dfn C, Dfn D, Dfn E, Dfn F, Dfn G, Dfn H, Dfn I, Dfn J, Dfn K, Dfn L, Dfn M, Dfn X, Dfn Y	53.3%
	Macrolactin	Mln A, Mln B, Mln C, Mln D, Mln E, Mln F, Mln G, Mln H, Mln I	88.9%

## References

[b1] Arguelles-Arias A. *et al.* *Bacillus amyloliquefaciens* GA1 as a source of potent antibiotics and other secondary metabolites for biocontrol of plant pathogens. Microb. Cell Fact. 8, 1–12 (2009). 19941639 10.1186/1475-2859-8-63 PMC2787494

[b2] Leclere V. *et al.* Mycosubtilin overproduction by *Bacillus subtilis* BBG100 enhances the organism’s antagonistic and biocontrol activities. Appl. Environ. Microb. 71, 4577–4584 (2005). 10.1128/AEM.71.8.4577-4584.2005 PMC1183317 16085851

[b3] Chen X. *et al.* Difficidin and bacilysin produced by plant-associated *Bacillus amyloliquefaciens* dare efficient in controlling fire blight disease. J. Biotechnol. 140, 38–44 (2009). 19061923 10.1016/j.jbiotec.2008.10.015

[b4] Senghor A., Liang W. J. & Ho W. C. Integrated control of *Colletotrichum gloeosporioides* on mango fruit in Taiwan by the combination of *Bacillus subtilis* and fruit bagging. Biocontrol Sci. Techn. 17, 575–580 (2007).

[b5] Yang Z., Guo H. & Zhang X. Study on the control of peach post harvest diseases using *Bacillus subtilis*. *China* Fruits 23, 35–38 (2008).

[b6] Kotan R., Dikbas N. & Bostan H. Biological control of post harvest disease caused by *Aspergillus flavus* on stored lemon fruits. Afr. J. Biotechnol. 8, 209–214 (2009).

[b7] Borriss R. *et al.* Relationship of *Bacillus amyloliquefaciens* clades associated with strains DSM 7T and FZB42T: a proposal for *Bacillus amyloliquefaciens* subsp. *amyloliquefaciens* subsp. nov. and *Bacillus amyloliquefaciens* subsp. plantarum subsp. nov. based on complete genome sequence comparisons. Int. J. Syst. Evol. Micr. 61, 1786–1801 (2011). 10.1099/ijs.0.023267-0 20817842

[b8] Yu G. Y., Sinclair J. B., Hartman G. L. & Bertagnolli B. L. Production of iturin A by *Bacillus amyloliquefaciens* suppressing *Rhizoctonia solani*. Soil Biol. Biochem. 34, 955–963 (2002).

[b9] Donadio S., Monciardini P. & Sosio M. Polyketide synthases and nonribosomal peptide synthetases: the emerging view from bacterial genomics. Nat. Prod. Rep. 24, 1073–1109 (2007). 17898898 10.1039/b514050c

[b10] Kopp F. & Marahiel M. A. Where chemistry meets biology: the chemoenzymatic synthesis of nonribosomal peptides and polyketides. Curr. Opin. Biotech. 18, 513–520 (2007). 17997093 10.1016/j.copbio.2007.09.009

[b11] Ongena M. & Jacques P. Bacillus lipopeptides: versatile weapons for plant disease biocontrol. Trends Microbiol. 16, 115–125 (2008). 18289856 10.1016/j.tim.2007.12.009

[b12] Ongena M. *et al.* Surfactin and fengycin, lipopeptides of Bacillus subtilis as elicitors of induced systemic resistance in plants. Environ. Microbiol. 9, 1084–1090 (2007). 17359279 10.1111/j.1462-2920.2006.01202.x

[b13] Raaijmakers J. M., De Bruijn I., Nybroe O. & Ongena M. Natural functions of lipopeptides from *Bacillus* and *Pseudomonas*: more than surfactants and antibiotics. *FEMS* Microbiol. Rev. 6, 1037–1062 (2010). 10.1111/j.1574-6976.2010.00221.x 20412310

[b14] Cochrane S. A. & Vederas J. C. Lipopeptides from *Bacillusand Paenibacillus* spp. : a gold mine of antibiotic candidates. Med. Res. Rev. 1, 4–31 (2016). 24866700 10.1002/med.21321

[b15] Weinert B. T. *et al.* Lysine succinylation is a frequently occurring modification in prokaryotes and eukaryotes and extensively overlaps with acetylation. Cell Rep. 4, 842–851 (2013). 23954790 10.1016/j.celrep.2013.07.024

[b16] Arif M., Selvi B. R. & Kundu T. K. Lysine acetylation: the tale of a modification from transcription regulation to metabolism. Chembiochem. 11, 1501–1504 (2010). 20578118 10.1002/cbic.201000292

[b17] Zhang K., Zheng S., Yang J. S., Chen Y. & Cheng Z. Comprehensive profiling of protein lysine acetylation in *Escherichia coli*. J. Proteome Res. 12, 844–851 (2013). 23294111 10.1021/pr300912q

[b18] Zhang J. *et al.* Lysine acetylation is a highly abundant and evolutionarily conserved modification in *Escherichia coli*. Mol. Cell Proteomics 8, 215–225 (2009). 18723842 10.1074/mcp.M800187-MCP200 PMC2634580

[b19] Kim D. *et al.* The acetylproteome of Gram-positive model bacterium *Bacillus subtilis*. Proteomics 13, 1726–1736 (2013). 23468065 10.1002/pmic.201200001

[b20] Okanishi H., Kim K., Masui R. & Kuramitsu S. Acetylome with structural mapping reveals the significance of lysine acetylation in *Thermus thermophilus*. J. Proteome Res. 12, 3952–3968 (2013). 23901841 10.1021/pr400245k

[b21] Lee D. W. *et al.* Proteomic analysis of acetylation in thermophilic *Geobacillus kaustophilus*. Proteomics 13, 2278–2282 (2013). 23696451 10.1002/pmic.201200072

[b22] Wu X. *et al.* Differential lysine acetylation profiles of *Erwinia amylovora* strains revealed by proteomics. J. Proteomics 79, 60–71 (2013). 23234799 10.1016/j.jprot.2012.12.001 PMC4418653

[b23] Henriksen P. *et al.* Proteome-wide analysis of lysine acetylation suggests its broad regulatory scope in *Saccharomyces cerevisiae*. Mol. Cell Proteomics 11, 1510–1522 (2012). 22865919 10.1074/mcp.M112.017251 PMC3494197

[b24] Xie L. X. *et al.* Proteome-wide lysine acetylation profiling of the human pathogen *Mycobacterium tuberculosis*. *Int. J. Biochem*. Cell B 59, 193–202 (2015). 10.1016/j.biocel.2014.11.010 25456444

[b25] Zhao S. *et al.* Regulation of cellular metabolism by protein lysine acetylation. Science 327, 1000–1004 (2010). 20167786 10.1126/science.1179689 PMC3232675

[b26] Huang D., Li Z. H., You D., Zhou Y. & Ye B. C. Lysine acetylproteome analysis suggests its roles in primary and secondary metabolism in *Saccharopolyspora erythraea*. Appl. Microbiol. Biot. 99, 1399–1413 (2015). 10.1007/s00253-014-6144-2 25487885

[b27] Liao G. J., Xie L., Li X., Cheng Z. & Xie J. Unexpected extensive lysine acetylation in the trump-card antibiotic producer *Streptomyces roseosporus* revealed by proteome-wide profiling. J. proteomics 106, 260–269 (2014). 24768905 10.1016/j.jprot.2014.04.017

[b28] Koumoutsi A. *et al.* Structural and functional characterization of gene clusters directing nonribosomal synthesis of bioactive cyclic lipopeptides in *Bacillus amyloliquefaciens* strain FZB42. J. Bacterial. 186, 1084–1096 (2004). 10.1128/JB.186.4.1084-1096.2004 PMC344220 14762003

[b29] Chen X. H. *et al.* Structural and functional characterization of three polyketide synthase gene clusters in *Bacillus amyloliquefaciens* FZB42. J. Bacterial. 188, 4024–4036 (2006). 10.1128/JB.00052-06 PMC1482889 16707694

[b30] Finking R. & Marahiel M. A. Biosynthesis of nonribosomal peptides 1. Annu. Rev. Microbiol. 58, 453–588 (2004). 15487945 10.1146/annurev.micro.58.030603.123615

[b31] Cane D. E. A special thematic issue on polyketide and nonribosomal polypeptide biosynthesis. Chem. Rev. 97, 2706 (1997). 10.1021/cr970097g 11851465

[b32] Butcher R. A. *et al.* The identification of bacillaene, the product of the PksX megacomplex in Bacillus subtilis. P. Natl. Acad. Sci. USA 104, 1506–1509 (2007). 10.1073/pnas.0610503104 PMC1785240 17234808

[b33] Roongsawang N., Washio K. & Morikawa M. Diversity of nonribosomal peptide synthetases involved in the biosynthesis of lipopeptide biosurfactants. Int. J. Mol. Sci. 12, 141–172 (2010). 21339982 10.3390/ijms12010141 PMC3039948

[b34] Zhu Z., Zhang F., Wei Z., Ran W. & Shen Q. The usage of rice straw as a major substrate for the production of surfactin by *Bacillus amyloliquefaciens* XZ-173 in solid-state fermentation. J. Environ. Manage. 127, 96–102 (2013). 23685270 10.1016/j.jenvman.2013.04.017

[b35] Kishore D. & Mukherjee A. K. Comparison of lipopeptide biosurfactants production by *Bacillus subtilis* strains in submerged and solid state fermentation systems using a cheap carbon source: Some industrial applications of biosurfactants. Process Biochem. 42, 1191–1199 (2007).

[b36] Khan A. W., Rahman M. S., Zohora U. S., Okanami M. & Ano T. Production of surfactin using pentose carbohydrate by *Bacillus subtilis*. J. Environ. Sci. 23, S63–S65 (2011). 10.1016/S1001-0742(11)61079-6 25084596

[b37] Oda K. *et al.* Proteomic analysis of extracellular proteins from *Aspergillus oryzae* grown under submerged and solid-state culture conditions. Appl. Environ. Microbiol. 72, 3448–3457 (2006). 16672490 10.1128/AEM.72.5.3448-3457.2006 PMC1472361

[b38] Pan J. *et al.* Systematic analysis of the lysine acetylome in *Vibrio parahemolyticus*. J. Proteome Res. 13, 3294–3302 (2014). 24874924 10.1021/pr500133t

[b39] Mo R. *et al.* Acetylome analysis reveals the involvement of lysine acetylation in photosynthesis and carbon metabolism in the model cyanobacterium *Synechocystis* sp. PCC 6803. J. Proteome Res. 14, 1275–1286 (2015). 25621733 10.1021/pr501275a

[b40] Ouidir T., Cosette P., Jouenne T. & Hardouin J. Proteomic profiling of lysine acetylation in *Pseudomonas aeruginosa* reveals the diversity of acetylated proteins. Proteomics 15, 2152–2157 (2015). 25900529 10.1002/pmic.201500056

