# Supplementary material for: Acetylome analysis reveals the involvement of lysine acetylation in biosynthesis of antibiotics in Bacillus amyloliquefaciens
Source: Sci Rep. 2016 Jan 29;6:20108. doi: 10.1038/srep20108 (PMC4731788; doi:10.1038/srep20108)
Supplement: Supplementary Figures [file srep20108-s1.doc]

**Acetylome analysis reveals the involvement of lysine acetylation in biosynthesis of antibiotics in** ***Bacillus amyloliquefaciens***

Lin Liu^1^, Guangyuan Wang^1^, Limin Song^2^, Binna Lv^2^, Wenxing Liang^2^*

^1^Shandong Province Key Laboratory of Applied Mycology, College of Life Sciences, Qingdao Agricultural University, Qingdao 266109, China. ^2^The Key Laboratory of Integrated Crop Pest Management of Shandong Province, College of Agronomy and Plant Protection, Qingdao Agricultural University, Qingdao 266109, China. Correspondence and requests for materials should be addressed to W.L. (wliang1@qau.edu.cn)

**Figure S1.** **Proteome-wide identification of lysine acetylation sites in** ***B. amyloliquefaciens*.** **a** Mass error distribution of all identified peptides. **b** Peptide length distribution.


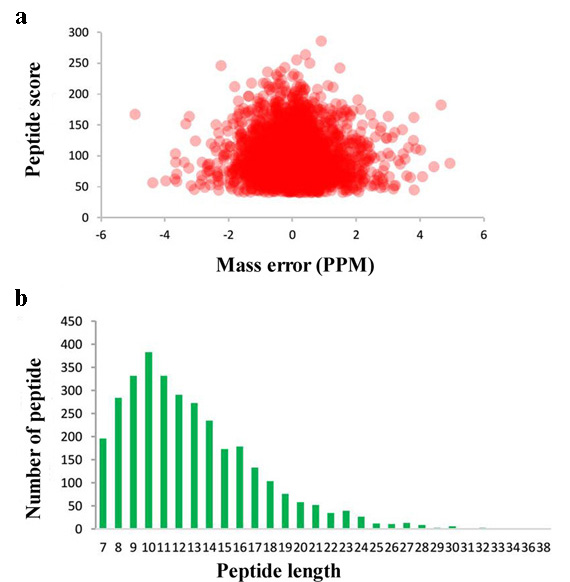


**Figure S2. KEGG pathway enrichment analysis of the acetylated proteins in ribosome.** The acetylated proteins are in yellow.


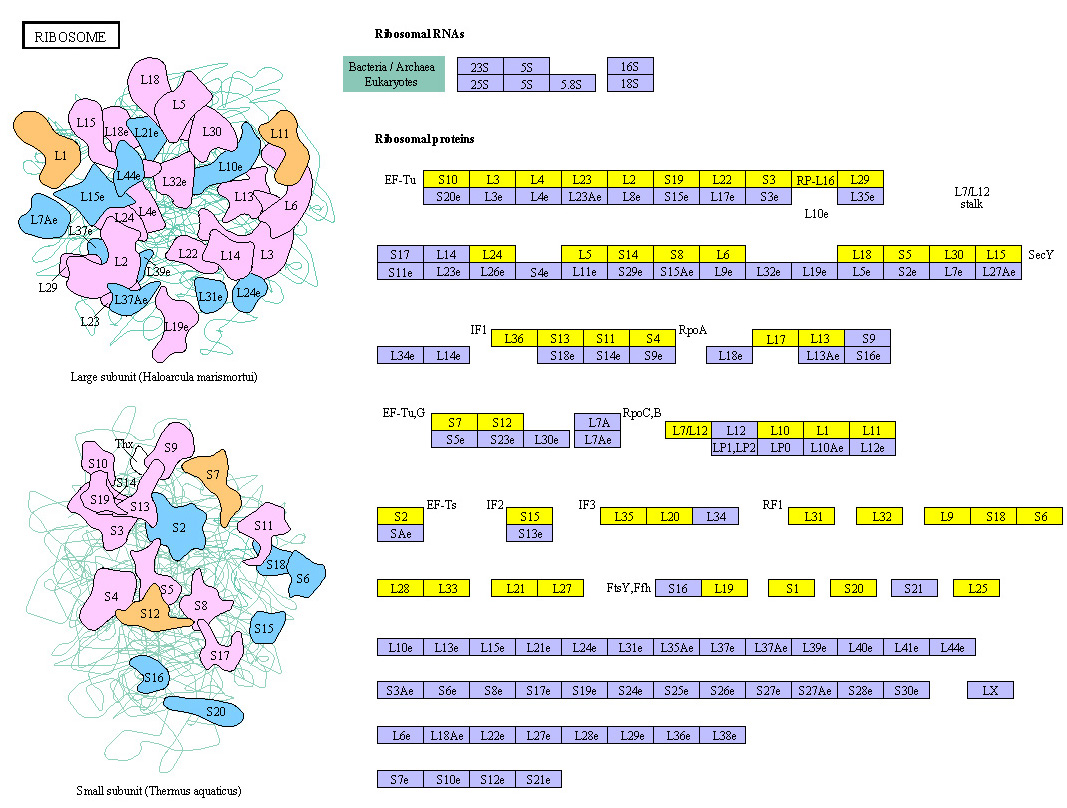


**Figure S3. KEGG pathway enrichment analysis of the acetylated proteins in aminoacyl-tRNA biosynthesis.** The acetylated proteins are in yellow.


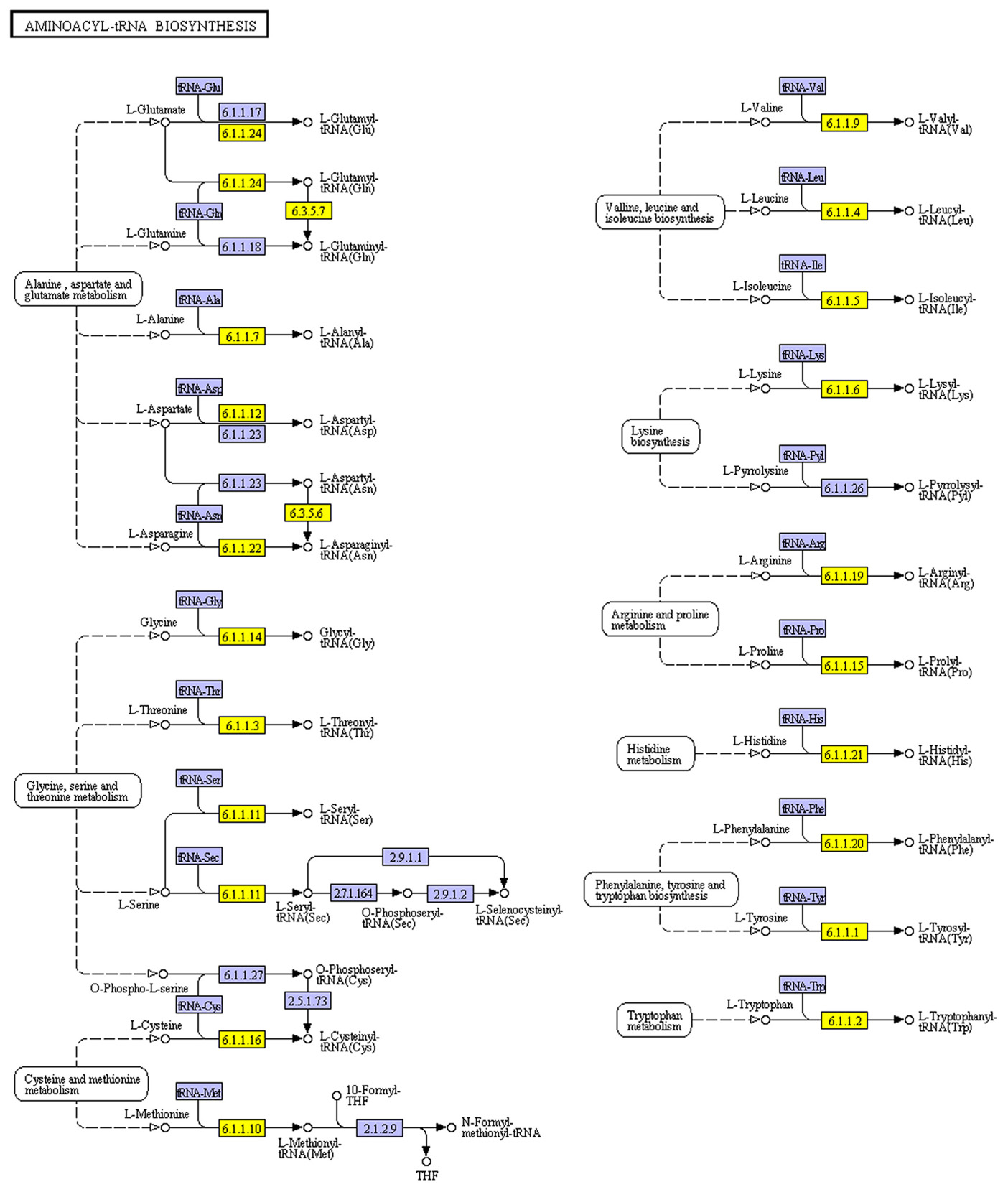


**Figure S4. KEGG pathway enrichment analysis of the acetylated proteins in purine metabolism.** The acetylated proteins are in yellow.


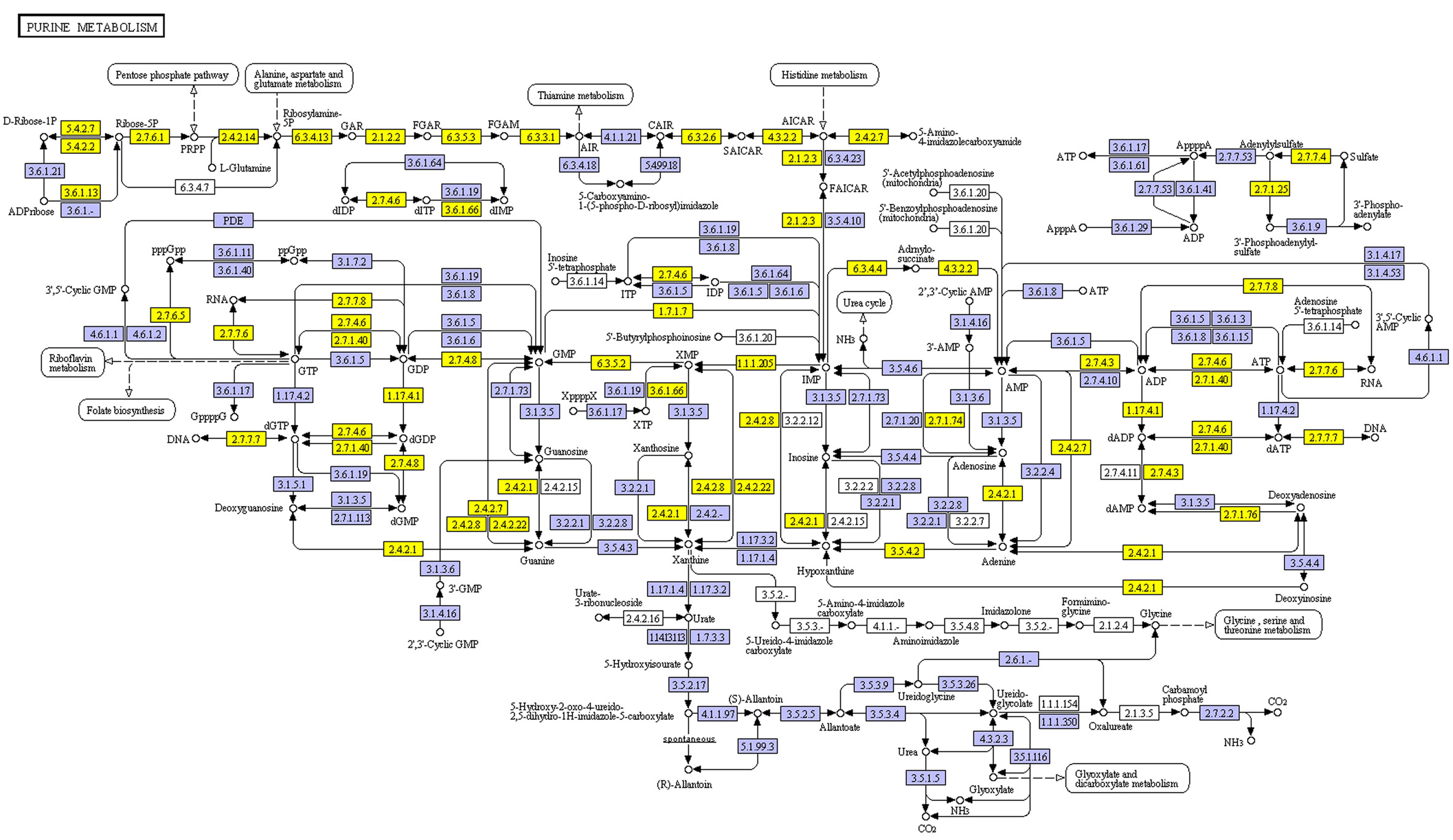


**Figure S5. KEGG pathway enrichment analysis of the acetylated proteins in pyrimidine metabolism.** The acetylated proteins are in yellow.

**
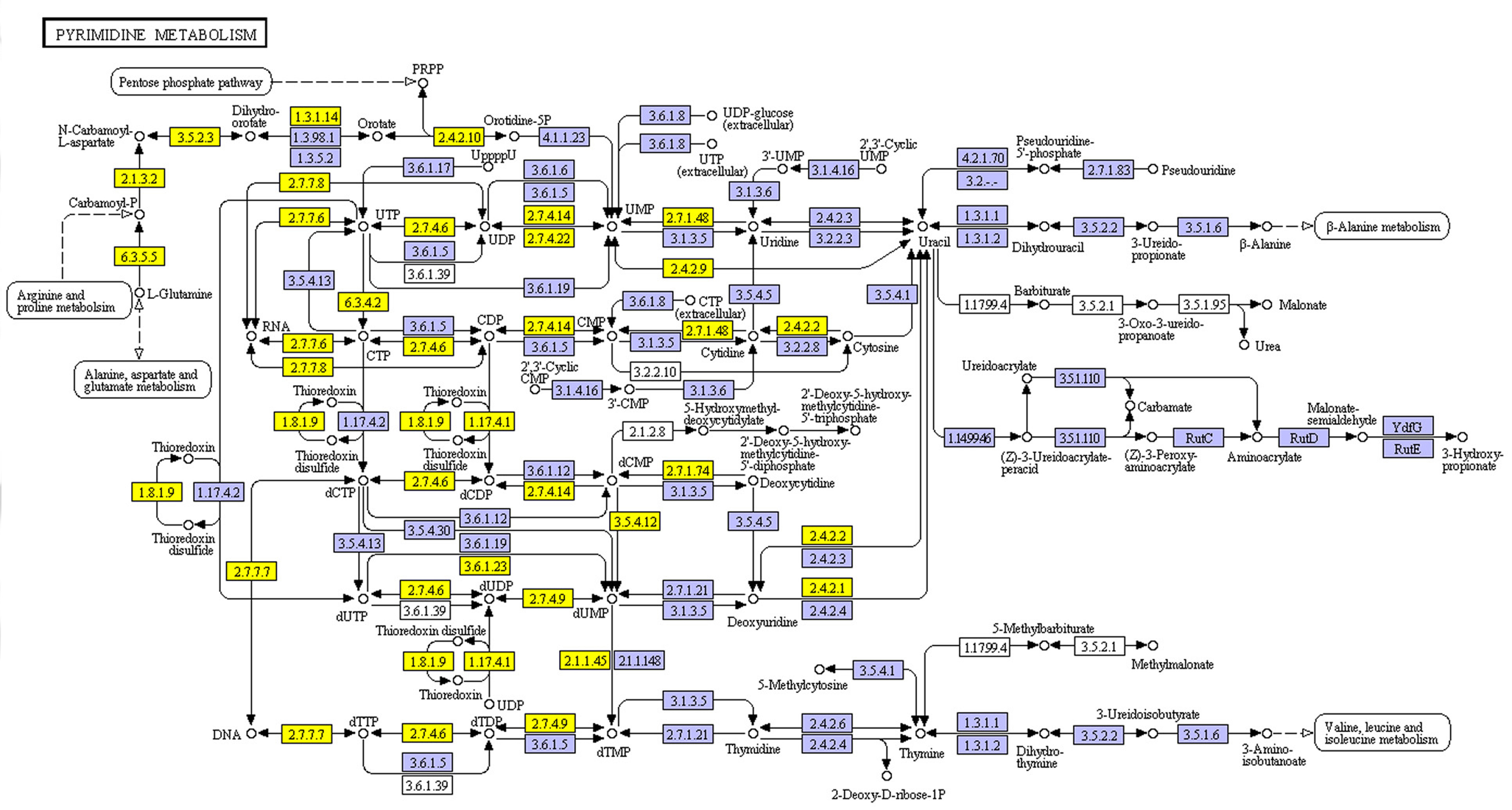
**
